# Visnagin: A novel cardioprotective agent against anthracycline toxicity (Review)

**DOI:** 10.3892/mi.2024.161

**Published:** 2024-05-13

**Authors:** Omar Obeidat, Ali Obeidat, Abedallah Obeidat, Mohamed F. Ismail

**Affiliations:** 1 Graduate Medical Education Program, College of Medicine, University of Central Florida, Orlando, FL 32816, USA; 2 Internal Medicine Residency Program, HCA Florida North Florida Hospital, Gainesville, FL 32605, USA; 3 Division of Cardiology, Department of Internal Medicine, Faculty of Medicine, Jordan University of Science and Technology, Irbid 22110, Jordan

**Keywords:** doxorubicin, cardiotoxicity, visnagin, cancer chemotherapy, cardioprotection, anthracycline

## Abstract

Doxorubicin (DOX), a cornerstone of cancer chemotherapy, is marred by its dose-dependent cardiotoxicity, leading to cardiomyopathy and heart failure. The epidemiology of DOX-related cardiotoxicity highlights its cumulative, progressive nature, with a significant impact on the health of patients. The pathophysiological mechanisms involve mitochondrial dysfunction, oxidative stress and disrupted calcium homeostasis in cardiomyocytes. Despite the search for effective cardioprotective strategies, current treatments offer limited efficacy. Visnagin emerges as a potential solution, known for its vasodilatory and anti-inflammatory properties, and recent studies suggest its cardioprotective efficacy against DOX-induced cardiotoxicity through mitochondrial protection, the modulation of key signaling pathways and the inhibition of apoptosis. The present review aimed to provide a comprehensive overview of the mechanisms of action of visnagin, as well as to provide experimental evidence, and potential integration into cancer treatment regimens, highlighting its promise as a novel therapeutic agent for managing cardiotoxicity in patients undergoing anthracycline chemotherapy.

## 1. Introduction

Doxorubicin (DOX), an anthracycline antibiotic, has been integral to cancer chemotherapy since the 1960s (1,2). It is highly effective against a wide range of cancers, including breast cancer, lymphomas and sarcomas (1,2). However, its significant drawback, dose-dependent cardiotoxicity, often offsets its oncological benefits. This cardiotoxicity, leading to irreversible cardiomyopathy and heart failure, severely affects the health and survival of patients (1,3,4).

The epidemiology of DOX-related cardiotoxicity is marked by its dose-dependent, cumulative and progressive nature (1,2). Anthracyclines such as DOX have historically been linked to cardiac complications, affecting up to 5% of patients with left ventricular dysfunction and heart failure (5). Reducing cumulative anthracycline doses helps lower the incidence of heart failure, with rates ranging from 3.5% at 400 mg/m^2^ to 18-48% at 700 mg/m^2^ (6). Globally, heart failure due to the use of anthracyclines accounts for 4.5-7% of cases; thus, this poses a significant concern alongside cancer (6,7). Monitoring and addressing this cardiotoxicity is essential for improving patient outcomes.

The cardinal mechanism of DOX-induced cardiotoxicity centers around mitochondrial dysfunction in cardiomyocytes (8). These heart muscle cells, heavily reliant on mitochondrial energy production, are particularly vulnerable to the effects of DOX on mitochondrial structure and function (8-10). This disruption results in the excessive generation of reactive oxygen species (ROS) and oxidative stress, key mediators of cardiac damage (10,11). Additionally, DOX disrupts calcium homeostasis within cardiac cells, further exacerbating the injury (9).

Over the past decades, the search for effective cardioprotective strategies against DOX-related cardiotoxicity has been challenging. The currently available treatments, such as dexrazoxane, offer limited efficacy and are associated with their own set of adverse effects (12-14). These limitations highlight the urgent need for more effective and safer cardioprotective interventions.

Visnagin, a furanochromone, is known for its vasodilatory and anti-inflammatory properties. It exhibits favorable pharmacokinetics with good bioavailability and a safety profile conducive to therapeutic use (15-19). Traditionally, visnagin has been used for its spasmolytic and diuretic effects (18,19). The exploration of its potential in cardioprotection is a recent development and represents a novel therapeutic application (20).

The present review aimed to provide a comprehensive overview of the cardioprotective properties of visnagin against DOX-induced cardiotoxicity. A summary of its molecular mechanisms is also presented, also providing experimental evidence from various studies, and discussing the potential for the integration of visnagin into cancer therapeutic regimens. The objective of the present review was to highlight visnagin as a novel therapeutic agent that could provide a safer and more effective approach for managing cardiotoxicity in patients undergoing anthracycline chemotherapy.

## 2. Molecular characteristics and biological activity of visnagin

**Figure 1:**
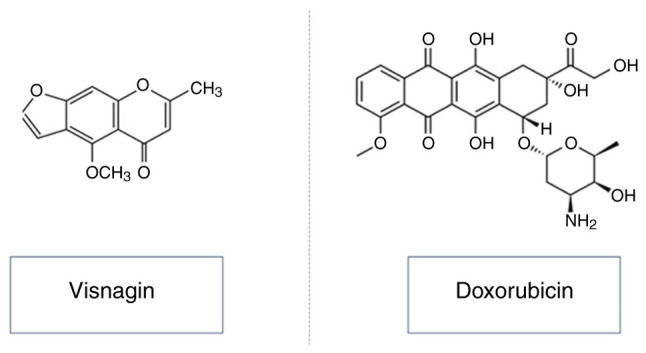
Chemical structure for visnagin and doxorubicin.

Visnagin, a bioactive compound found in the fruit of *Ammi visnaga*, belongs to the furanochromone class of natural substances and possesses a distinctive molecular structure characterized by a chromone backbone fused with a furan ring (21) (chemical structure presented in Fig. 1). This unique structure incorporates a methoxy group at the 5-position and a methyl group attached to the furan ring, contributing to its pharmacological activity (21). While the pharmacokinetic properties of visnagin are not extensively detailed in the existing literature, its pharmacodynamics are noteworthy.

Visnagin exhibits vasorelaxant properties, primarily affecting vascular smooth muscle cells, leading to a reduction in blood pressure without significantly altering the heart rate. Additionally, visnagin mildly affects cardiac contractility, with a modest impact on heart muscle function (22). Furthermore, visnagin exhibits a capacity to modulate mitochondrial enzymes and attenuate oxidative stress, thereby conferring protection to cardiac tissue (23). Beyond its cardiovascular effects, visnagin has been shown to be associated with a spectrum of pharmacological activities, encompassing antispasmodic, antidiabetic potential, anti-inflammatory, antimicrobial, cytotoxic, antioxidant and immunostimulatory properties (18). Furthermore, visnagin displays promise in addressing hair loss, exerts antimutagenic effects, and displays activity against larvae and weeds, thereby suggesting herbicidal potential (18).

## 3. Safety and side-effects

To the best of our knowledge, there are limited data available regarding the safety profile of visangin. While some potential side-effects have been reported in the literature, such as nausea, dizziness and constipation, the evidence supporting these claims not substantial (17). No specific contraindications have been thoroughly established, apart from a strong advisement against its use during pregnancy due to the uterine stimulant activity of its active constituent, khellin (17,18). Additionally, while there are no formal warnings for the dried drug or its preparations, it is advisable to avoid direct sunlight exposure during treatment with khella to prevent photosensitivity. This is of particular importance when handling the fresh plant, as its sap contains photosensitizing agents that can affect various organs. High doses of visangin have been found to be associated with increases in the levels of liver enzymes, indicating potential liver damage, thus underscoring the necessity for careful management of dosage and treatment duration. Further research is warranted to study the safety and side-effects profile of this compound (17,18).

## 4. Multifaceted mechanisms of cardioprotection of visnagin

Visnagin has been extensively explored as a potential cardioprotective agent in the context of myocardial infarction (MI) and cardiotoxicity induced by various agents. The majority of the current understanding of the mechanisms of visnagin stems from animal models, particularly utilizing rats and zebrafish (22-24). The use of the zebrafish model is critical in cardiotoxicity research due to its genetic and physiological parallels with humans. Its transparent embryos enable the direct observation of cardiovascular dynamics, greatly advancing the comprehension of cardiac pathologies. Additionally, the rapid development, prolific reproduction, and amenability to large-scale genetic and pharmacological studies render zebrafish an excellent model for high-throughput drug screening, particularly in the quest for treatments against DOX-induced cardiotoxicity (24-26).

Studies utilizing zebrafish models, notably by those by Liu *et al* (25) and Asnani *et al* (26), have identified visnagin as a promising candidate for preventing DOX-related cardiotoxicity. These models have demonstrated that treatment with visnagin results in reduced cardiotoxicity and in improved overall cardiac function. A key mechanism proposed involves the interaction of visnagin with mitochondrial components. DOX is known to impair mitochondrial metabolism, significantly contributing to cardiotoxicity (8-10). Visnagin appears to safeguard mitochondrial integrity and function, maintaining mitochondrial membrane potential and inhibiting apoptosis initiation, which is typically triggered by DOX-induced oxidative stress (23). A specific interaction has been noted between visnagin and malate dehydrogenase (MDH2), an enzyme critical in the tricarboxylic acid cycle (25,26). MDH2 operates in concert with the malate-aspartate shuttle (MAS), which is pivotal in transferring reducing equivalents into mitochondria for oxidation. The interaction between visnagin and MDH2, and thereby MAS, may mirror the cardioprotective effects observed in ischemic preconditioning by modulating mitochondrial respiration (27).

Furthermore, Asnani *et al* (26) uncovered another potential cardioprotective mechanism via the inhibition of cytochrome P450 1A (CYP1A). CYP1A inhibition has been linked to cardioprotection, suggesting a potential role in modulating DOX-induced cardiotoxicity (25,28). DOX has been shown to directly inhibit cytochrome P450 2J2, an enzyme integral in the heart for producing cardioprotective epoxyeicosatrienoic acids (EETs) (29,30). This inhibition leads to a reduction in EETs, exacerbating cardiotoxicity. By contrast, non-cardiotoxic analogues of DOX do not significantly affect EET production, despite competitive inhibition (29,30).

The relevance of these findings is underscored by the high degree of homology between zebrafish and human enzymes in the MAS pathway, particularly MDH2 and aminoaspartate transferase (31). This similarity suggests a conservation of metabolic pathways across species. Moreover, in contrast to cardiac cells, tumor cells predominantly utilize aerobic glycolysis (the Warburg effect), indicating that MAS inhibition could protect against cardiotoxic injury without substantially affecting tumor metabolism (32).

In rat models, visnagin has been shown to mitigate damage from MI and isoproterenol-induced MI, suggesting broader cardioprotective effects (33). The anti-inflammatory and antioxidant properties of visnagin have been noted to reduce cardiac injury post-isoproterenol exposure. It modulates key molecular pathways, such as NF-κB, TNF-α, IL-6 and PPARγ, and upregulates nuclear factor erythroid 2-related factor 2/heme oxygenase-1 signaling. These effects suggest a direct consequence of suppressed ROS generation and enhanced antioxidant defenses, crucial in mitigating oxidative damage, inflammation and apoptosis (19,33).

Another study using rats highlighted the role of visnagin in enhancing autophagy in ischemic cardiac areas during ischemia/reperfusion injury (34). This process is vital for cardiomyocyte survival, promoting protein homeostasis and mitigating mitochondrial damage. Visnagin has also been observed to reduce apoptosis in ischemic areas, potentially through modulation of the aryl hydrocarbon receptor (AHR) signaling pathway (34,35).

The intravenous administration of visnagin during reperfusion has been found to significantly reduce the myocardial infarct size, and mitigate cardiac dysfunction and fibrosis (34). Additionally, the vascular effects of visnagin, including the relaxation of blood vessels and the inhibition of cyclic nucleotide phosphodiesterase isoenzymes, further contribute to its cardioprotective profile (18). In addition, it has also been reported that visnagin can reduce the levels of pro-inflammatory cytokines, such as IL-1, IL-6 and TNF-α (25).

In the realm of oncology, the effects of visnagin on cancer cells have been noted. Research has demonstrated its ability to inhibit the proliferation of malignant cells, such as in melanoma and hepatocellular carcinoma, by inducing ROS production and activating pro-apoptotic pathways, and by modulating AHR signaling (18,36-38).

**Figure 2:**
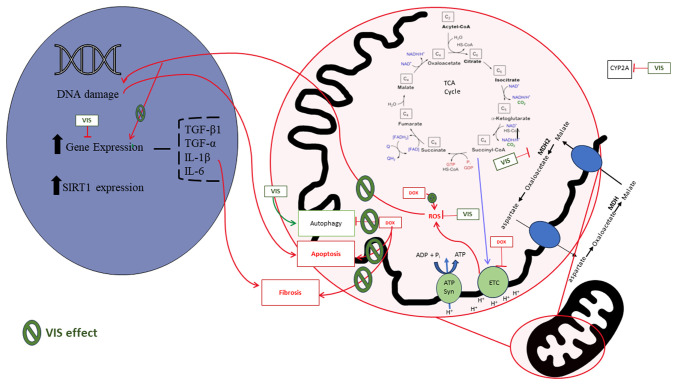
Schematic diagram illustrating the mechanisms of cardiotoxicity of DOX and cardioprotection of visnagin. DOX, doxorubicin; ETC, electron transport chain; ATP Syn, ATP synthase; ROS, reactive oxygen species; MDH, malate dehydrogenase; VIS, visnagin; SIRT1, sirtuin 1; TCA, tricarboxylic acid; CYP2A, cytochrome P450 2A.

In summary, the growing body of evidence points to visnagin as a multifaceted agent with potential cardioprotective effects mediated through various molecular pathways, as summarized in Fig. 2. Its interaction with mitochondrial enzymes, the modulation of key signaling pathways, and effects on autophagy and apoptosis underscore its potential as a therapeutic agent in cardioprotection, particularly in the context of DOX-induced cardiotoxicity.

## 5. Challenges and future directions

In order to facilitate the translation of visnagin from laboratory research to clinical applications, a detailed, phased clinical trial strategy is proposed. Initially, double-blinded, placebo-controlled studies need to be conducted using murine models to establish the safety, pharmacokinetics and pharmacodynamics of visnagin. As previously demonstrated in the study by Liu *et al* (25), it is suggested that visnagin be administered at a dose of 25 mg/kg, dissolved in a vehicle consisting of 10% ethanol and 90% olive oil, visnagin will be delivered intravenously, immediately followed by a contralateral intravenous injection of DOX. This stage is critical for validating preliminary efficacy and safety. Upon the successful completion of animal trials, the study of visnagin will progress to human trials, commencing with a small cohort to refine safety profiles and optimal dosing parameters. These initial human trials should focus on key clinical endpoints such as cardiac remodeling, ejection fraction, symptomatology and the overall quality of life. Secondary outcomes will assess the impact on the efficacy of cancer therapy and patient mortality. As safety and efficacy data accumulate, the trials should be expanded to include a larger group of participants, ensuring a comprehensive assessment of the therapeutic viability of visnagin and its integration into current cancer treatment paradigms. This structured approach is designed to rigorously test and validate the cardioprotective effects of visnagin in oncological settings.

The exploration of visnagin as a cardioprotective agent against DOX-induced toxicity heralds a potential paradigm shift in the management of cancer therapy-related cardiac complications. The multifaceted mechanisms by which visnagin offers protection to the heart underline its unique therapeutic potential. From preserving mitochondrial integrity to modulating key enzymes and cellular processes, visnagin stands as a beacon of hope in mitigating the deleterious effects of one of the most potent chemotherapeutic agents.

However, the road to integrating visnagin into clinical practice is fraught with challenges. These challenges not only encompass the scientific and clinical rigor needed to substantiate the efficacy of visnagin, but also involve a broader understanding of its long-term implications in the treatment of cancer. The pursuit of this endeavor is not merely an academic exercise, but a crucial step towards improving the outcomes and quality of life of patients.

In the realm of oncology, where the balance between therapeutic efficacy and adverse effects is delicate, visnagin could potentially redefine treatment paradigms. Its ability to safeguard the heart while allowing effective cancer treatment presents a dual benefit, addressing a longstanding issue in cancer therapy. Future research into visnagin, therefore, is not just a pursuit of a new therapeutic agent, but a quest to redefine the boundaries of cancer treatment, where patient survival is complemented by an improved quality of life.

The comprehensive investigation of the cardioprotective properties of visnagin, from molecular mechanisms to clinical applications, is a testament to the evolving nature of cancer therapy. As further insight is provided into the intricate interplay between cancer treatment and its systemic effects, agents such as visnagin emerge as harbingers of a new era in oncology, an era where treatment efficacy and patient safety are not mutually exclusive, but are synergistically achieved. The journey of visnagin from a traditional remedy to a potential cornerstone in cancer therapy encapsulates the essence of translational medicine, bridging the gap between bench and bedside.

The present review has certain limitations, which should be mentioned. The present review predominantly utilizes data from zebrafish models, which, while invaluable for providing initial mechanistic insight, may not fully predict the therapeutic outcomes in humans due to physiological and metabolic differences. Additionally, the absence of human clinical trials in the literature reviewed constitutes a significant limitation, as these studies are crucial for determining the safety, efficacy and appropriate dosages of visnagin in human populations. Furthermore, the present review could benefit from a broader scope that includes investigations into potential drug-drug interactions, particularly concerning concurrent cancer therapies that patients may be undergoing. Another limitation is the lack of longitudinal studies to assess the long-term effects and sustainability of the cardioprotective benefits of visnagin, which are essential for evaluating its practical application in chronic conditions such as cancer. Addressing these gaps through comprehensive clinical trials and expanded research frameworks is essential for advancing the translational journey of visnagin from bench to bedside.

In conclusion, the journey of visnagin from a traditional remedy to a potential game-changer in oncology underscores the dynamic nature of medical research and patient care. Its role in the future of cancer therapy remains to be fully realized; however, the promise it holds is undeniable. As science forges ahead in the quest for safer and more effective cancer treatments, visnagin stands at the forefront, symbolizing hope, innovation and the relentless pursuit of excellence in healthcare.

## Data Availability

Not applicable.
